# The Retroverted Uterus and Pelvic Floor Dysfunction: 400 BC to 2025 AD

**DOI:** 10.1007/s00192-025-06167-1

**Published:** 2025-06-11

**Authors:** Bernard T. Haylen, Dzung Vu

**Affiliations:** 1 University of New South Wales, Kensington, N.S.W Australia; 2 Notre Dame University, Darlinghurst, Sydney, N.S.W Australia; 3 Coogee, Australia

**Keywords:** Defecatory dysfunction, Pelvic floor dysfunction, Retroverted uterus, Uterine/pelvic organ prolapse, Uterine retroversion, Voiding dysfunction

## Abstract

**Introduction and Hypothesis:**

In 2025, the retroverted uterus will be more formally recognized with a section in the Female Reproductive System Chapter of Gray’s Anatomy. This study examines all available publications to develop a detailed history and associations with pelvic floor dysfunction.

**Methods:**

Medline and Embase databases extending back indefinitely were searched looking for references on the retroverted uterus or uterine retroversion. The limited number of articles relevant to any specific section prevented the development of specific selection criteria or the construction of tabulation.

**Results:**

From 400 BC to 2025 AD, a total of 308 publications were able to be sourced, of which 50 were pre-1900. Obstetric indications accounted for 116 (37.7%) publications, nearly all incarceration of a retroverted gravid uterus. Gynaecological indications, including conservative and surgical interventions, accounted from 107 (34.7%) publications. Factors relevant to pelvic floor dysfunction, including imaging, diagnosis and prevalence, were generally in the remaining 85 (27.6%) publications.

**Conclusions:**

The retroverted uterus has a long, rich and interesting history, with significant interruptions in reporting. The most relevant classification is anatomical according to the presence or absence of retroversion and whether retroflexion of the uterine fundus is additionally present. Its aetiology is more likely to be developmental, with a limited acquired component. Although there is a familial tendency, genetic studies have been inconclusive.

Prevalence is 16–18% (1:6) women, increasing in the presence of pelvic floor dysfunction.

The most significant gynaecological association is with uterine/pelvic organ prolapse and some types of vaginal prolapse. The literature has countless case reports of both obstetric (particularly incarceration) and gynaecological episodes of acute urinary retention. Less dramatic, chronic, sometimes cyclical symptoms of voiding and defecatory dysfunction, as well as pelvic pain, have also been recorded in publications.

Uterine retroversion is most commonly asymptomatic, requiring no treatment. Symptomatic cases, including a prolapsed retroverted uterus, may, at times, require surgical relief.

## Introduction

In 2025, the retroverted uterus will be more formally recognized with a section in the Female Reproductive System chapter (78) of Gray’s Anatomy [1]. It will be referred to as an “anatomical variant”. The retroverted uterus has not yet been the subject of a full and timeless literature review to establish its history, possibly over millennia, and its interrelations with conclusions relevant to the area of pelvic floor dysfunction. The authors felt it to be appropriate timing to perform such a study.

## Materials and Methods

All available articles or references to the search terms of “retroverted uterus” or “uterine retroversion” in the Medline and Embase databases extending back indefinitely were examined, as well as other references on the retroverted uterus derived from those or other sources. The search was not restricted, though mainly English-language publications were reviewed.

The limited number of articles relevant to any specific section prevented the development of specific selection criteria or the construction of tabulation.

A wide range of aspects related to the retroverted uterus were explored as a result of the above search including definition, history (in different groups of years), aetiology, clinical diagnosis, ultrasound diagnosis, prevalence, relationship to pelvic organ prolapse and other “most common” urodynamically based diagnoses [2], impact clinically on voiding and defecatory dysfunctions, conservative therapies, as well as arguments for surgery and the nature of the potential surgeries.

## Results

From 400 BC to 2025 AD, a total of 308 publications were sourced, of which 50 were pre-1900. Obstetric indications accounted for 116 publications (37.7%), nearly all on incarceration of a retroverted gravid uterus. Gynaecological indications, including conservative and surgical interventions, accounted from 107 publications (34.7%). Factors relevant to pelvic floor dysfunction, including imaging, diagnosis and prevalence, were in the remaining 85 publications (27.6%).

### Definition

The definition and simple classification of the retroverted uterus has been non-controversial over the last 240 years [3–7]. A uterus is described as retroverted when the cervix is pointing downwards and forwards and the axis of the body of the uterus is directed backwards, towards the hollow of the sacrum, and away from its “normal” (anteverted) position overlying the bladder. The longitudinal axis of the anteverted uterus is approximately at right angles to the vagina, whereas the axis of the retroverted uterus tends to be similar (closer to parallel) to that of the vaginal axis. The anteriorly placed cervix is close to the bladder and urethra, as opposed to the position of the cervix of an anteverted uterus lying in the posterior fornix and directed infero-posteriorly.

**Fig. 1:**
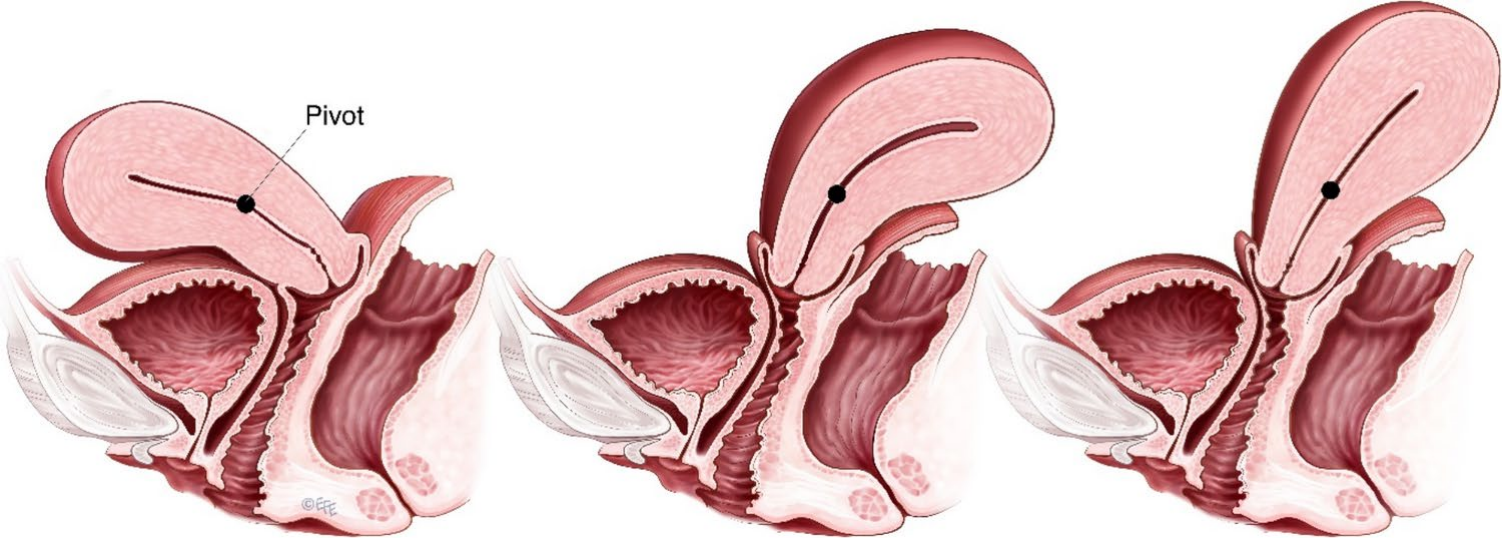
Left: anteverted uterus. Right: retroverted uterus. Centre: retroverted, retroflexed uterus.(Copyright: Bernard Haylen and Levent Efe, leventefe.com.au)

If angulation of the body (corpus) of the uterus on the cervix, at the level of the isthmus, is more pronounced, the retroverted uterus should additionally be termed “retroflexed” (see Fig. 1).

### History

The retroverted uterus has a long and interesting history, with large gaps in reporting. The majority of the case reports are obstetric cases, in particular the incarceration of a gravid retroverted uterus in the second trimester (generally) causing urinary retention and, at times, maternal and foetal compromise.

#### A: Early History (400 BC to 1890 AD)—Relationship to (Clinical) Voiding and Defecatory Dysfunction; Early Prevalence Figures 47 out of 308 Publications (15.3%)

The retroverted uterus is mentioned in the writings of Hippocrates (around 400 BC) according to Smith in his 1869 paper [8], specifically the books “De Morbis Mulierum” and “De Naturâ Muliebri”. Edwards [9], in 1849 cites references to the retroverted uterus by Philomenas, amongst other “ancients” such as Rod à Castro, Mauricean and La Motte. The description given by Aëtius Tetrabiblos (Medice Artis Principes, fol. 1567) and recorded in the original Latin by Smith [8], points to an author (Aëtius) clearly acquainted with different forms of malpositioning of the uterus. The recommended treatment of acute severe cases (urinary retention, obstructed defecation) by urinary catheter, enema and pressure on the uterine fundus per rectum, the latter measures (as recorded by Smith), “delegated to his midwife”, is not so different from equivalent current emergency treatments.

Following Aëtius Tetrabiblos, Edwards [9] suggested that the subject of the retroverted uterus had been “almost lost sight of” until Desgranges (1715), Gregoire (1746) and Hunter (1754) drew attention to it and “placed the matter in a clear and intelligent light”.

The celebrated anatomist, William Hunter (1718–1783) is generally credited with being the first to have named “retroversion of the uterus” and described several case histories [10]. Some of the cases in this and an earlier paper were communicated to Hunter by William Bird (1750) and Maxwell Garthshore (1732–1812). Cockell [7] also refers to Hunter’s 1954 lecture on the subject. Edwards [9] also cites the clinical entity of the retroverted uterus as being described by John Maubray (died 1732) in his popular text, the “Female Physician” (London, 1724).

##### Voiding Dysfunction

Including urinary retention in pregnancy, most of the reported cases are due to a uterus impacted (“incarcerated”) in the pouch of Douglas, with corresponding marked cervical pressure anteriorly and superiorly placed on the bladder base. Oliver [11], in 1890, reported a case of death from rupture of the bladder and subsequent peritonitis in a fixed retroverted gravid uterus. In another case described by Corkhill [12] in 1887, the cervical os was palpable above the pelvic brim. Edwards [9], in 1849, reported three cases of voiding dysfunction associated with non-gravid uterine retroversion, with two of the uteri being “hypertrophied” (perhaps with fibroid involvement).

##### Defecatory Dysfunction

**Fig. 2:**
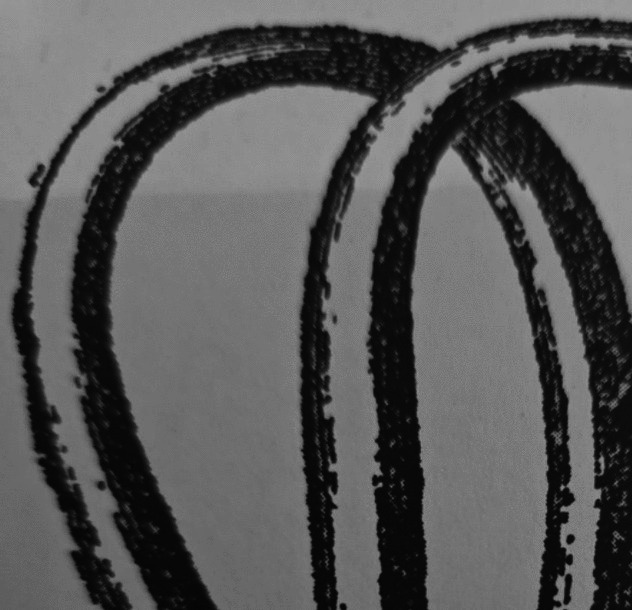
An early pessary. From W. Murray (1868) [16]. A spring pessary for the relief of retroversion and retroflexion of the uterus by active extra-uterine support. Permission granted to reproduce by Elsevier

Herman [13, 14], in 1882, highlighted the impact of a severely retroverted and retroflexed uterus on the rectum, citing a case from the *Edinburgh Medical and Surgical Journal* of 1854 (p. 336) in which the fundus protruded at the anus when the patient defecated. Edwards [9], in 1854, noted that the pressure of the fundus against the rectum can produce “severe constipation” or even “difficulty in going to stool at all”. Cyclical (premenstrual—when the uterus is more congested and thus heavier) exacerbation of defecatory dysfunctions, due to retroversion, recognized as far back as 1854 [9], most likely represents a degree of extrinsic impingement on the rectum. Herman [13, 14], in 1882, discussed the relative merits of a Hodge and ring pessary in relieving symptoms, with support from Smith [8] and subsequent support from McCann [15] and Luker [4]. There were some more unusual designs, such as the one used by Murray [16] in 1868 (Fig. 2), which might be directed at supporting the lateral fornices of the vaginal vault [1] to elevate the uterus.

##### Early Prevalence Figures

Herman [13, 14] quotes the extensive clinical (clearly no ultrasound imaging available) studies of Vedeler (*Archiv fur Gynäkologie*, vol xix, p. 294), a physician who practiced in Christiania (more likely the old name for Oslo, Norway, though possibly a district in Copenhagen, Denmark). The overall prevalence of retroversion in 1504 healthy women was 16.5%: 14% in healthy virgins (414 examined); 12% in healthy nullipara (506 examined); 22% in parous women (584 examined). Of 1158 women with uterine symptoms, 259 (22.4%) had a retroverted uterus.

#### Period B: (1891–1955)—More Emphasis on Clinical Diagnosis, Conservative and Surgical Management; 37 out of 308 Publications (12.0%)

Luker [4] emphasizes in 1934 that most retroverted uteri are asymptomatic, with women generally unaware of their presence; no investigation or treatment is required. Surgical correction (ventrosuspension) was becoming popular in this period, generally by a modified Gilliam ventrosuspension (a.k.a. ventrofixation). A permanent suture (silk at the time) was used to attach a loop of round ligament on each side to the rectus sheath. Luker [4], however, emphasized that “it is preferable to give a good trial to medical treatment (including pessaries) before resorting to a surgical operation” for symptomatic retroversion. Indications for surgical relief of uterine retroversion in 1934 [4] included:

1. Infertility (sterility and miscarriage—now a rare reason
2. Fixed retroversion associated with pelvic pain (of whatever cause)
3. Dyspareunia
4. Pelvic floor discomfort
5. Voiding and defecatory dysfunction

Rendle-Short [17], in 1941, quotes “only” a 50% perfect symptomatic and anatomical result from a series of 120 patients who had undergone ventrosuspension, with other functional or orthopaedic causes possibly present.

Attempts at further clinical classifications during this time [4, 13–15] did not last. As previously noted, the simplest and most relevant clinical classification is anatomical, depending on the presence or absence of retroversion and whether retroflexion is additionally present.

Fothergill [18] perhaps too closely links retroversion with the development of prolapse in his 1912 comments: “every case of classical prolapse is characterized by both cystocoele and retroversion”. The Fothergill (Manchester) repair involves anterior colporrhaphy, plication of the merged uterosacral and cardinal ligaments, and amputation of the cervix.

#### Period C: (1956–1985) 41 out of 308 Publications (13.3%)

There was a slowing down in the performance of ventrosuspensions; this was a relatively quiet period in the history of the retroverted uterus, although there was some discussion on aetiology [5].

The retroverted uterus is thought normally to be a developmental occurrence [6] although acquired retroversion can occur owing to the effects of endometriosis, pelvic inflammatory disease and pelvic tumours. There is no clear evidence to support a “traditional theory” [18] that the anteverted uterus becomes retroverted as it prolapses. Symmonds [5] proposes a strong argument, on the basis of the axes of the retroverted uterus and the vagina being in line: “intra-abdominal pressure can exert a piston-like action on the retroverted uterus driving it down the vagina”. In contrast, the anteverted uterus would be forced infero-posteriorly, receiving support from the rectum.

#### Period D: (1986–2007)—Transvaginal Ultrasound Clarifies the Diagnosis and Prevalence of the Retroverted Uterus and Relationship to Pelvic Organ Prolapse; 93 out of 308 Publications (30.2%)

The advent of transvaginal ultrasound in urogynecology in 1987 allowed a far more accurate appreciation of the retroverted uterus than had previously been possible. Apart from measuring bladder volumes [19] including postvoid residuals, some of its other routine uses were identifying intercurrent pelvic/lower urinary tract pathology and the presence/absence of uterine retroversion (empty bladder important) [19, 20].

Freimanis and Jones [20] determined that an accurate ultrasound of the retroverted uterus required

1. The use of transvaginal ultrasound because of the superior resolution of that modality and the proximity of the probe to the area of interest
2. An empty bladder

In confirming these criteria, Haylen et al. [21] noted a significant reduction in the prevalence of the retroverted uterus in 480 general gynaecology patients receiving subspecialist ultrasound assessment from 18% (empty bladder, transvaginal ultrasound used) to 13% (full bladder, transabdominal ultrasound used).

In a study of a urogynaecological patient population [7] of 592 women using a clinical and transvaginal ultrasound (empty bladder) diagnosis, a 34% prevalence of the retroverted uterus, 79% greater than for the general gynaecological population, was found, with uterine (and co-existent vaginal) prolapse issues the likely reason for this 1:3 prevalence. Fauconnier et al. [22] noted a 24% prevalence of the retroverted uterus by clinical examination alone (ultrasound was not routinely used) in an unselected population of women attending for routine gynaecological assessment. In the same study, women with retroversion were noted to have a significantly higher prevalence of dyspareunia and severe dysmenorrhoea (67% vs 42%; both symptoms, *p* = 0.03), with no association with noncyclic, ovulation or premenstrual pain.

In a further analysis of the earlier urogynaecological patient population [7, 23] of 592 patients, using routine clinical and transvaginal ultrasound (empty bladder) diagnosis, there was a 4.5-fold increase in presentations with stage II–IV uterine prolapse in women with a retroverted uterus than in those with an anteverted uterus. There was a higher prevalence of stage II–III cystocoele (×1.9) and at least stage I vaginal vault descent (×4.7) in association with a retroverted uterus than was the case with an anteverted uterus. The earlier comments of Symmonds [5] provide a very plausible explanation.

#### Period E: (2008–2025)—Relationship of the Retroverted Uterus to Other “Most Common” Urodynamic Diagnoses; Repopularization of Ventrosuspension by Laparoscopy; 90 out of 308 Publications (29.2%)

The advent of the International Urogynecological Association/International Continence Society Female Pelvic Floor Terminology Report for pelvic floor dysfunction [2] allowed the analysis of any relationships between the retroverted uterus and the five “most common” urodynamically based diagnoses (apart from pelvic organ prolapse): urodynamic stress incontinence (USI); detrusor overactivity (DO); bladder oversensitivity (BO); recurrent urinary tract infections (UTIs); voiding dysfunction.

##### Urodynamic Stress Incontinence and the Retroverted Uterus

In the previously cited studies [7, 23], there were no significant differences in the prevalence of USI between women with

1. An anteverted uterus
2. A retroverted uterus
3. An absent uterus

##### Detrusor Overactivity, Bladder Oversensitivity and the Retroverted Uterus

No significant relationship was found between the presence or absence of a retroverted uterus and DO or BO diagnoses [23, 24].

##### Recurrent Urinary Tract Infection and the Retroverted Uterus

Theoretically, the chances of recurrent UTI (three or more medically diagnosed episodes in 12 months) might be more likely in women with uterine retroversion owing to more likely impingement on the anteriorly placed cervix and, in turn, greater frictional pressure of the cervix on the bladder base. In a large study, using the modified criteria of two or more medically diagnosed UTIs in 12 months, no significant relationship was found between recurrent UTI and uterine retroversion (*p* = 0.366). In this and the above studies, we have found no other studies to contradict the stated findings [23, 25].

##### Retroverted Uterus and Voiding Dysfunction

Voiding dysfunction has been defined as abnormally slow and/or incomplete micturition as determined by an abnormally slow urine flow rate and/or an abnormally high postvoid residual [2]. Despite case reports of acute and chronic retention of urine associated with a retroverted uterus, there is no significant relationship between the retroverted uterus and an increasing postvoid residual (*p* = 0.163). In a urogynaecological patient population [23], no significant differences were found between women with an anteverted uterus, those with a retroverted uterus and those with an absent uterus in relation to the diagnosis of voiding dysfunction (previously termed voiding difficulty). In a further study [26], the absence of a significant relationship between voiding dysfunction and the retroverted uterus was confirmed (*p* = 0.250).

##### Re-popularization of Ventrosuspension by Laparoscopy

Of the 183 papers in this and the previous time period, 31 (16.9%) focused on laparoscopic ventrosuspension. Newer and less morbid techniques for laparoscopic ventrosuspension, combined with a renewed awareness of the possible adverse effects of uterine retroversion for pelvic floor/general gynaecological indications, has seen a re-invigorated interest in surgical management. The non-fertility indications alluded to in 1934 [4], namely fixed retroversion associated with pelvic pain (of whatever cause), dyspareunia, pelvic floor discomfort, and voiding and defecatory difficulties, are still present. In a more general or endoscopic gynaecological practice, pelvic pain (including dysmenorrhoea and dyspareunia) may be the more predominant symptoms. Evidence for the beneficial effect of laparoscopic ventrosuspension has been recently cited [27], although the absence of randomized clinical trials precludes acceptance of uterine suspension as an established therapy for pelvic pain [27]. Multiple other scenarios for laparoscopic ventrosuspension have been quoted [28–31].

## Conclusions

The retroverted uterus has a long, rich and interesting history, with significant interruptions in reporting. The most relevant contemporary classification is anatomical according to the presence or absence of retroversion and whether retroflexion is additionally present. Its aetiology is more likely developmental (with a possible familial component, clinically though not genetically proven at this stage), with a limited acquired component.

Most retroverted uteri are asymptomatic, with women unaware of their presence; no investigation or treatment is required. The literature is rich in acute obstetric and gynaecological emergencies, the majority involving urinary retention, whereby surgical management might, at times, be required. The non-acute clinical diagnosis involves slightly non-specific symptoms, though more defined signs, with the most accurate diagnosis involving the use of transvaginal ultrasound with the bladder empty. Its prevalence is approximately 16–18%, or 1 in 6 women, increasing significantly in the presence of symptoms of pelvic floor dysfunction.

The main significant pelvic floor dysfunction association is with uterine and, to a degree, certain forms of vaginal prolapse, with no significant association with other diagnoses of pelvic floor dysfunction. As noted, episodes of acute urinary retention in all retroverted uterine scenarios have been reported, with chronic, often cyclical, symptoms of voiding and defecatory dysfunction also recorded.

Where symptoms are sufficiently problematic, conservative measures (pessaries [4, 12, 13] and physical therapy) can be trialled. Pessaries thought to provide apical support (Gellhorn, ring and cube) are preferable. Surgical management is generally by ventrosuspension (non-prolapse) or pelvic floor repair (prolapse). Most current ventrosuspensions involve the round ligaments (commonly shortening by concertina/pleating to elevate the uterus); however, attachment to the anterior abdominal wall may still be used. Although standardization of techniques and specific success rates using that technique were not a feature of the literature search, studies quoting a 50% long-term anatomical and symptomatic success rate were noted [17, 31].
